# Calcined Chitosan-Supported Layered Double Hydroxides: An Efficient and Recyclable Adsorbent for the Removal of Fluoride from an Aqueous Solution

**DOI:** 10.3390/ma10111320

**Published:** 2017-11-17

**Authors:** Hanjun Wu, Huali Zhang, Qingxue Yang, Dongsheng Wang, Weijun Zhang, Xiaofang Yang

**Affiliations:** 1 Faculty of Materials Science and Chemistry, China University of Geosciences, Wuhan 430074, China; wuhj1204@cug.edu.cn (H.W.); yangqingxue1014@163.com (Q.Y.); wgds@rcees.ac.cn (D.W.); 2 School of Chemistry and Environmental Engineering, Wuhan Institute of Technology, Wuhan 430074, China; zhanghl413@126.com; 3 State Key Laboratory of Environmental Aquatic Chemistry, Research Center for Eco-Environmental Sciences, Chinese Academy of Sciences, Beijing 100085, China; 4 School of Environmental Studies, China University of Geosciences, Wuhan 430074, China

**Keywords:** chitosan, layered double hydroxides, adsorption, fluoride removal, regeneration

## Abstract

In this work, calcined chitosan-supported layered double hydroxides (CSLDO) were synthesized through a co-precipitation method that restrained the particles’ aggregation of LDHs and exhibited huge specific surface areas, which can enhance the fluoride adsorption capacity. CSLDOs were characterized by physical and chemical methods and used for fluoride adsorption in an aqueous solution. The results indicated that the nanoparticles were constructed first and then assembled to form a porous and layered structure, and chitosan-supported layered double hydroxides (CSLDHs) calcined at 400 °C (CSLDO400) showed the highest specific surface area of 116.98 m^2^·g^−1^ and the largest pore volume of 0.411 cm^3^·g^−1^. CSLDO400 exhibited excellent adsorption performance at a wide pH range from 5 to 9 for fluoride. The adsorption kinetics indicated that the adsorption reached equilibrium after 120 min, and followed a pseudo-first-order model. It agreed well with the Langmuir isotherm with maximum adsorption amounts of 27.56 mg·g^−1^. The adsorption of fluoride ions was spontaneous and endothermic. Furthermore, CSLDO400 showed a high stability for fluoride removal; it could still achieve 68% removal for fluoride after repeating five times of adsorption–desorption cycles. This study demonstrated that CSLDO400 is a promising functional material to remove fluoride from surface/ground water.

## 1. Introduction

Excessive fluoride (F^−^) in groundwater is a serious problem worldwide [1]. Serious fluoride poisoning occurs frequently in many parts of the world, particularly in north and northeast China, Mexico, India, and Africa [2]. It is estimated that 200 million people still rely on groundwater with fluoride concentrations above the World Health Organization (WHO) guideline value (1.5 mg·L^−1^) [3]. Thus, water treatment for fluoride removal is extremely important in water purification. In order to remove fluoride from an aqueous solution, several processes such as adsorption, ion exchange, precipitation, and membrane techniques have been established [4,5,6,7]. Among these technologies, adsorption is a widely used technique for fluoride removal from water because the operating procedure is simple. A wide variety of adsorbents have been used for the removal of fluoride from water, such as carbonaceous materials [8], or solid industrial wastes like red mud, fly ash [9], activated and impregnated alumina [10], and layered double hydroxides (LDHs) [11]. However, traditional adsorption materials showed low removal efficiency in actual drinking water treatment. This is because the adsorption capacity of most absorbents was greatly influenced by the fluoride concentration; it will significantly decrease with the decrease in fluoride concentration [12]. Contemplating all this, it is necessary to develop novel and effective materials for fluoridated water treatment.

In recent years, layered double hydroxides (LDHs) have drawn attention for the removal of various harmful anions and surfactants because they are eco-friendly materials [13,14,15,16]. They are well known as a class of synthetic anionic clay consisting of positively charged hydroxy layers of bivalent and trivalent metal ions [17]. The metal hydroxide can adsorb some anions through electrostatic interactions because these hydroxides are always positively charged [18]. The general formula representing LDHs is [M_x_^2+^M_y_^3+^(OH)_2(x+y)_]·A_y/n_^n−^·mH_2_O (M^3+^: trivalent metal ions, M^2+^: bivalent metal ions, A: exchangeable anion) [19]. LDHs have been studied as potential adsorbents (Zn–Al LDH and Mg–Al LDH) for removing fluoride ions from aqueous systems [11,20,21]. Most of these LDHs focus on aluminum-based compounds, and treat water with high fluoride concentrations. However, when using these materials in drinking water treatment, long-term exposure to Al has been pointed out as a potential risk factor for human health and the environment, and simultaneously results in a smaller adsorption capacity for low fluoride concentration. Considering that, LDHs are required to be modified for practical application. As modified LDHs, the calcined chitosan-supported Mg–Fe LDHs, which have never been studied, are environmentally friendly materials and may be applied in the removal of both high- and low-fluoride concentration water.

Chitosan (CS) is a biopolymer with a linear polysaccharide based on a glucosamine unit. As a cheap and environmentally friendly polymer material, CS has been widely used to adsorb a wide variety of organic pollutants due to the presence of active amino (–NH_2_) and hydroxyl (–OH) functional groups [22,23]. Taking into account the use in different forms, from powder or beads to film types, CS-based adsorbents are versatile materials. Chitosan–Fe^3+^ has been reported to remove alkaline dye from an aqueous solution as an adsorbent in the literature [24]. Thus, it is possible to make a chitosan–Fe^3+^ precursor and then synthesize chitosan-supported layered double hydroxides by the co-precipitation method. The calcined chitosan-supported layered double hydroxides may restrain the aggregation of LDHs and exhibit huge specific surface areas, which probably contributes to more adsorption sites and enhances the fluoride adsorption capacity.

Herein, we prepared a biodegradable, low-cost material that can act as a support for LDH and also enhance its fluoride adsorption capacity. Batch adsorption experiments were carried out to optimize the adsorption parameters; adsorption kinetics, isotherms and thermodynamics were also examined. In addition, the reusability of CSLDO was performed and evaluated.

## 2. Results and Discussion

### 2.1. Characterization of the Adsorbent

#### 2.1.1. Field Emission Scanning Electron Microscope (FEI-SEM) Analysis

**Figure 1:**
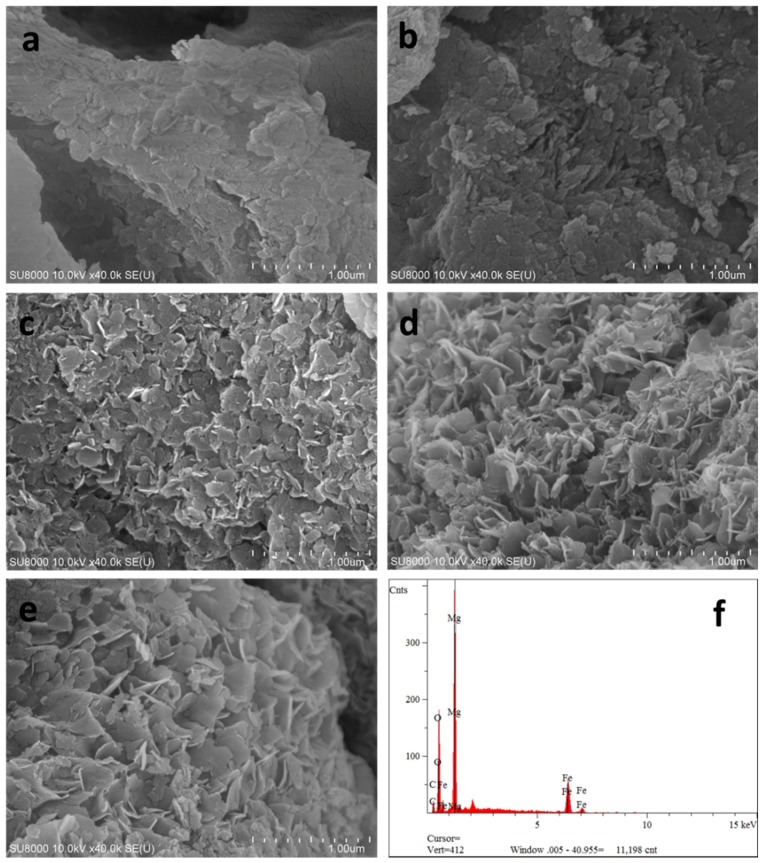
Scanning electron microscopic image of CSLDHs (**a**); LDO400 (**b**); CSLDHO300 (**c**); CSLDO400 (**d**); CSLDO500 (**e**); and the EDS of CSLDO400 (**f**).

FE-SEM was applied to observe the microstructure of the samples prepared by different methods. Representative micrographs of CSLDHs, LDO400, CSLDO300, CSLDO400, and CSLDO500 are presented in Figure 1a–e respectively. It can be seen from Figure 1a that the morphology of CSLDHs was dominated by mutual cross-linked lamellar structures. This phenomenon was attributed to the formation of LDHs crystal on the surface of chitosan–Fe^3+^. As shown in Figure 1b, the layered structure of LDO400 was obvious, and its surface was smooth. The micrographs of the calcined chitosan-supported LDHs are shown in Figure 1c–e. It can be seen that the CSLDOs had an irregular layered structure. Compared to the micrographs of CSLDHs and LDO400, the layered structure of CSLDO300, CSLDO400, and CSLDO500 was rougher; this might be because of the formation of holes in chitosan carbonization. As seen from an EDS analysis of CSLDO400 (Figure 1f), it was composed of Fe, Mg, O, and C. The products contained magnesium oxide and iron oxide with atomic ratios of around 3:1 for Mg/Fe, which was in accordance with the mole ratios for Mg(NO_3_)_2_/Fe(NO_3_)_3_ in the synthesis of CSLDO. The presence of C was likely to be caused by chitosan carbonization. It should be noted that the layer structure might play an important role in contaminant adsorption.

#### 2.1.2. BET Analysis

**Figure 2:**
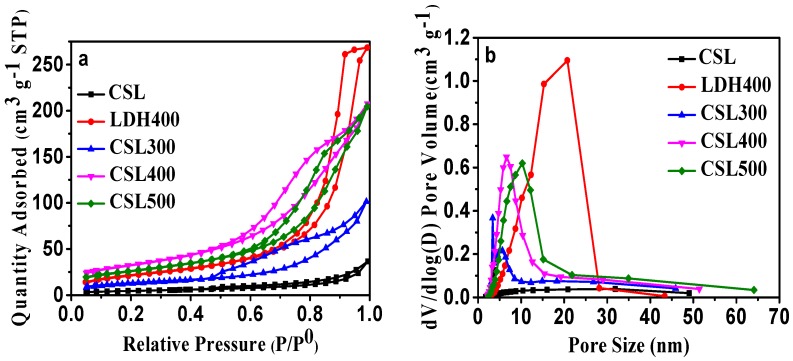
(**a**) N_2_ adsorption–desorption isotherms of CSLDHs, LDO400, CSLDO300, CSLDO400, and CSLDO500 at 77 K; and (**b**) pore size distribution.

The literature reported that the calcination of LDHs often produces very reactive mixed oxides [25,26]. Additionally, thermal activation at moderate temperatures can produce a high specific surface area and a high degree of microporosity. The porous properties of CSLDHs, LDO400, CSLDO300, CSLDO400, and CSLDO500 were analyzed by N_2_ adsorption–desorption isotherms at 77 K. As can be seen in Figure 2a, a high uptake of nitrogen was observed at low relative pressures, representing the microporous nature of the layered oxides and carbide networks. The isotherms were on a steep upward-sloping trend at higher pressures (P/P_0_ > 0.9), which might be ascribed to the presence of larger pores [27]. From the pore size distribution cures (Figure 2b), the five samples showed the predominant presence of mesopores.

Using the Barrett–Joyner–Halenda (BJH) and Brunauer–Emmett–Teller (BET) methods, the pore size distribution and specific surface areas of the samples were determined, respectively. The detailed results from the pore structure and surface area studies are summarized in Table 1. Obviously, the pore volume and surface area of CSLDO400 were larger than those of CSLDHs, LDO400, CSLDO300, and CSLDO500, while the pore size was smaller. This means that CSLDO400 has larger pore structure than that of the other samples. This phenomenon might be due to the special pore structure of the samples. During the calcination process, the carbonated chitosan support layer formed a carbonaceous porous carrier; the layered structure of hydrotalcite will be destroyed through the breakup of the crystal structure, resulting in the formation of a porous structure in the interlayer of CSLDO400. The total pore volumes of CSLDHs, LDO400, CSLDO300, CSLDO400, and CSLDO500 were calculated as 0.056, 0.323, 0.155, 0.412, and 0.318 cm^3^·g^−1^, respectively, and the pore diameters were 13.28, 15.83, 12.01, 8.839, and 10.99 nm (the so-called “mesoporous” size). It is generally accepted that the microstructure of adsorbents has an important effect on its water treatment performance, high specific surface area and mesoporous pores were favorable for higher adsorption capacity, indicating that CSLDO400 might be more effective for contaminant removal.

#### 2.1.3. FT-IR Analysis

**Figure 3:**
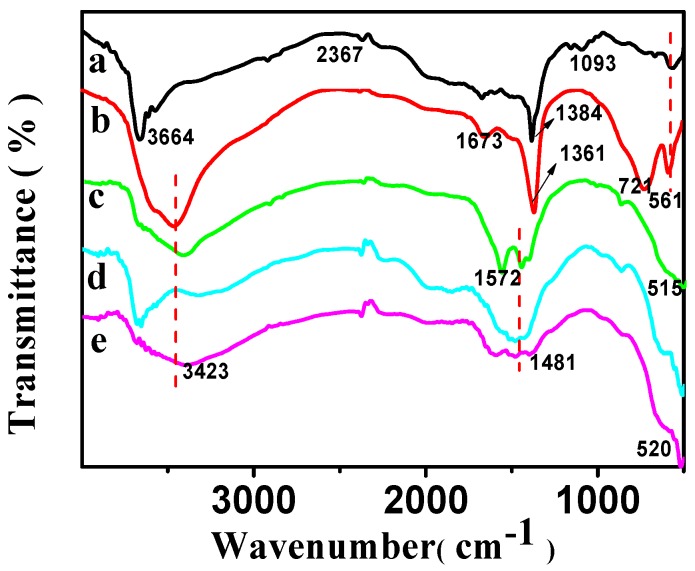
FT-IR spectra of CSLDHs (**a**); LDO400 (**b**); CSLDO300 (**c**); CSLDO400 (**d**); and CSLDO500 (**e**).

The FT-IR spectra of CSLDHs, LDO400, CSLDO300, CSLDO400, and CSLDO500 are displayed in Figure 3. As shown in Figure 3a, the two adsorption peaks at 3700–3500 cm^−1^ were related to N–H stretching vibration for the as-prepared sample (CSLDHs). This suggests that the LDHs are supported by chitosan, which contains amino group. Bands of NO_3_^−^ stretching at ~1384 cm^−1^ were derived from the interlayer ions of CSLDHs. The spectrum of the LDO400 (Figure 3b) was very similar to the spectrum of CSLDHs, except for the bands at 3700–3500 cm^−1^ and 1000–500 cm^−1^. However, it is important to mention that the band at 1000–500 cm^−1^ is slightly broader than that of the CSLDHs. This might be attributed to the peak overlapping of the interlayer anions and the carbonation of chitosan. The spectra of the LDO400 (Figure 3b), CSLDO300 (Figure 3c), CSLDO400 (Figure 3d), and CSLDO500 (Figure 3e) are very similar, and the bands at ~1440 and 1384 cm^−1^ derived from the interlayer anions (CO_3_^2−^ and NO_3_^−^) were not found. As is well known, those interlayer anions, especially carbonate ions, take up the adsorption sites of hydrotalcite because they have a strong affinity for hydrotalcite. It can be speculated that the calcination reduced interlayer anions, which may have a significant influence on the fluoride removal efficiency [12].

#### 2.1.4. XRD Analysis

**Figure 4:**
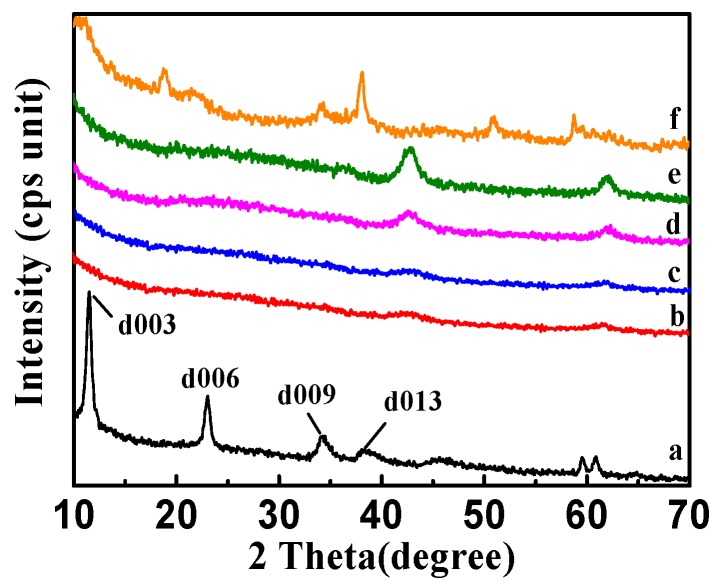
X-ray diffraction (XRD) patterns of CSLDHs (**a**); LDO400 (**b**); CSLDO300 (**c**); CSLDO400 (**d**); CSLDO500 (**e**); and regenerated CSLDO400 (**f**) after five adsorption–desorption cycles.

The X-ray diffraction (XRD) patterns of CSLDHs, LDO400, CSLDO300, CSLDO400, and CSLDO500 are shown in Figure 4. As seen from the synthesized products of CSLDHs in Figure 4a, symmetrical and sharp peaks appeared, indicating the highly crystalline nature of the samples. Meanwhile, the presence of 003, 006, and 009 diffraction peaks confirmed that synthesized products showed a typical and well-ordered structure of layered double hydroxides [28]. The patterns of calcined samples (Figure 4b–e) indicated that the diffraction peaks of layered double hydroxides have disappeared, the layered structure was destroyed, the crystal structure was changed, and only magnesium and iron oxide peaks were retained. As seen from the regenerated CSLDO400 in Figure 4f, the presence of 009 and 013 diffraction peaks indicated that its original layered hydrotalcite-like structure was reconstructed by the intercalation of fluoride ions into the interlayer region after adsorption. Yet, compared to CSLDHs, it can also be seen that the peaks of calcined products became broader and the intensity of the peaks decreased. This may be attributed to the formation of amorphous mixed oxides. LDHs may be changed into binary oxides after being calcined at a certain temperature, and restored to their original layered structure after entering into the water environment. This process is called “memory effect” [12], which is a significant characteristic of LDHs. Therefore, based on the “memory effect,” the calcined chitosan-supported LDHs may be forced to adsorb anions from the water environment.

### 2.2. Evaluation of Fluoride Removal Efficiency by the Prepared LDHs

#### 2.2.1. Effect of Calcinations on F^−^ Removal

**Figure 5:**
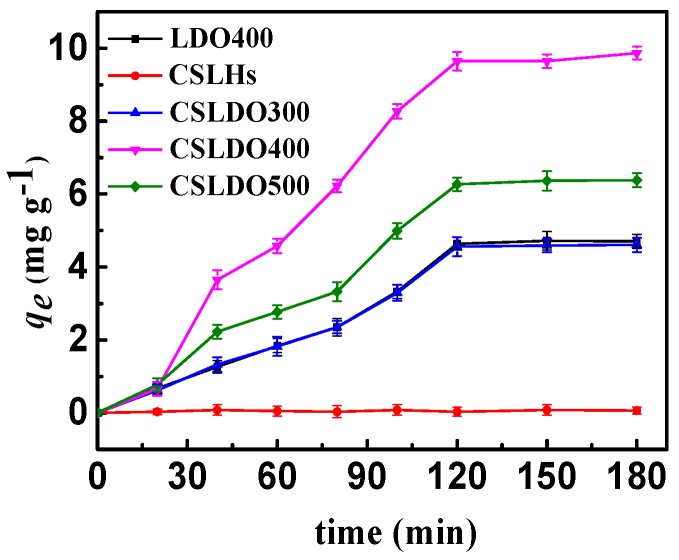
Adsorption capacities of CSLDHs, LDO400, CSLDO300, CSLDO400, and CSLDO500 to fluoride at different times (Adsorption dose, 0.625 g·L^−1^; solutions concentration, 10 mg·L^−1^; pH = 7; adsorption time, 3 h; temperature, 298 K).

As shown in Figure 5, the samples without calcination treatment (CSLDHs) showed very limited adsorption capacity *q_e_* (mg·g^−1^) for fluoride; the fluoride removal efficiency was significantly enhanced after calcination treatment. Moreover, the adsorption capacity of chitosan-supported double hydroxides reached the maximum at the calcination temperature of 400 °C. Meanwhile, the adsorption capacity of calcined chitosan-supported double hydroxides was higher than that of the unsupported ones. Since the affinities of various anions toward interlayers of hydrotalcite (LDHs) follow the order CO_3_^2−^ > SO_4_^2−^ > OH^−^ > F^−^ > Cl^−^ > Br^−^ > NO_3_^−^ > I^−^ [21], carbonate and hydroxyl ions are difficult to replace with fluoride ions toward interlayers of CSLDHs, which were not calcined. With the calcination temperature increasing, interlayer anions (carbonate and hydroxyl ions) were released gradually, and magnesium ion mixed oxides partially formed under calcination treatment at 400 °C. CSLDHs calcined at 500 °C showed smaller pore volume and surface area, and led to the formation of stable phases of MgFe_2_O_4_ spinel and MgO, so that the layered hydrotalcite-like structures could not be reconstructed. As mentioned above, the pore volume and surface area of CSLDO400 were larger than those of CSLDHs, LDO400, CSLDO300, and CSLDO500, which might contribute to more surface active adsorption sites and consequently higher fluoride removal efficiency. The results indicated that the BET surface area played an important role in the fluoride adsorption process. In summary, CSLDO400 was the optimal adsorbent for the following adsorption experiments.

#### 2.2.2. CSLDO400 Dosage

**Figure 6:**
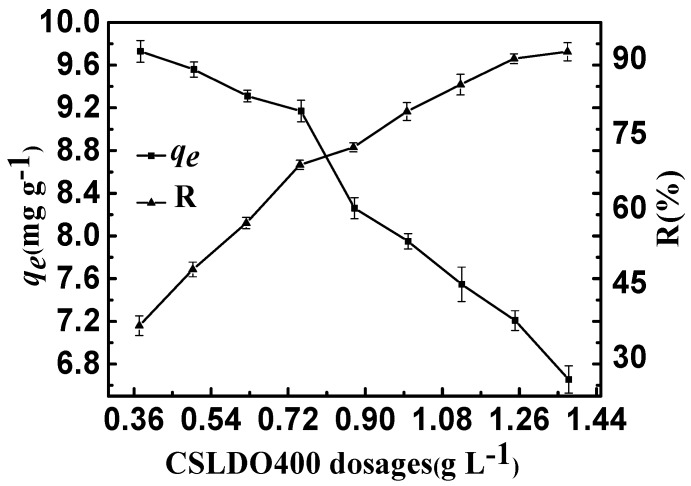
Effect of CSLDO400 dosages on the adsorption of fluoride (Solution concentration, 10 mg·L^−1^; pH = 7; adsorption time, 3 h, temperature, 298 K).

The effect of CSLDO400 dosage on fluoride removal is shown in Figure 6. It can be seen that the equilibrium removal rates (R: %) increased but the equilibrium adsorption amounts *q_e_* (mg·g^−1^) declined observably when the CSLDO400 dosage increased. The equilibrium removal rate (R: %) was almost unhanged when the dosage increased to above 1.25 g·L^−1^. However, the equilibrium adsorption amounts *q_e_* (mg·g^−1^) still decreased, maybe due to excessive CSLDO400 in aqueous solution. The enhancement of removal efficiency can be attributed to the high number of unsaturated adsorption sites, and an increase in the adsorption surface area and surface energy of CSLDO400. On the one hand, an increase in the concentration of CSLDO400 particles improved the chance of collision and agglomeration of CSLDO400 particles and the specific surface area decreased significantly. On the other hand, the absorbents had extra surface active sites because of more CSLDO400. Lastly, the adsorption capacity decreased because of the reduction of surface energy.

#### 2.2.3. Effect of pH

**Figure 7:**
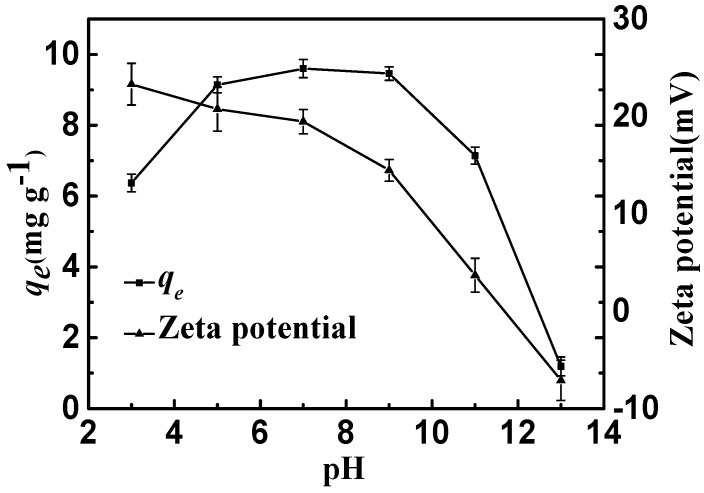
Adsorption capacities and zeta potential of CSLDO400 as a function of pH (Adsorption dose, 0.75 g·L^−1^; solutions concentration, 10 mg·L^−1^; adsorption time, 3 h; temperature: 298 K).

As shown in Figure 7, the adsorption equilibrium amounts of CSLDO400 for fluoride were almost unchanged when the initial pH values of the solutions ranged from 5 to 9, indicating that CSLDO400 performed well at fluoride removal at typical water pH, while it sharply decreased when the initial pH values ranged from 9 to 13. As shown in Figure 7, the zeta potential of CSLDO400 was reduced from 23.3 mV at pH 3 to −7.1 mV at pH 13, and isoelectric points appeared. This phenomenon might be due to the positive surplus charges, which were generated by the formation of an LDH structure (replacing some of the divalent cations by trivalent cations) [18]. Normally, the anions were first adsorbed on the surface and edge of the adsorbent by electrostatic effects and then exchanged with the interlayer anions of hydrotalcite-like compounds [29]. As shown in Figure 7, at low pH (pH < 9), CSLDO400 was always positively charged, which might be helpful for electrostatic interaction. It is well known that the adsorbent surface was able to be protonated because of the hydroxyl group on the adsorbent surface [30]; the surface charge was generated as follows [31]:

(1) $$\mathrm{MO}+H_{2}O↔{\mathrm{MOH}}_{2}^{+}+{\mathrm{OH}}^{-}$$

(2) $$\mathrm{MOH}+H_{2}O↔{\mathrm{MO}}^{-}+H_{3}O^{+}$$

(3) $$\mathrm{MO}+H_{2}O↔{\mathrm{MOH}}_{2}^{+}+{\mathrm{OH}}^{-}\overset{①\;X^{m-}}{↔}{\mathrm{MOH}}_{2}\cdots X+{\mathrm{OH}}^{-}+\overset{②\;X^{m-}}{↔}\mathrm{MX}+H_{2}O+{\mathrm{OH}}^{-}.$$

M and X^m−^ represent the metal element and the fluoride or anions, respectively; m indicates the valence of anions. At a low pH value, protonation promoted the formation of positively charged MOH^2+^ groups. Meanwhile, anions are adsorbed on the surface and then enter into the interlayer of the adsorbent due to the electrostatic attraction (Reaction ①) and ion exchange interaction (Reaction ②) (Equation (9)). Moreover, at high pH levels, hydroxyl showed a strong competitive adsorption effect on fluoride ions because hydroxyl has a similar radius of fluoride ion, resulting in a decrease in fluoride [32]. However at low pH, HF and H_2_O are dissociation of H^+^ and F^−^. As HF is a weakly ionized substance, the reaction of HF and double oxides caused damage to the double oxide structure. Therefore, pH 7 (range from 5 to 9) was maintained in the adsorption experiments.

#### 2.2.4. Effect of Co-Anions

**Figure 8:**
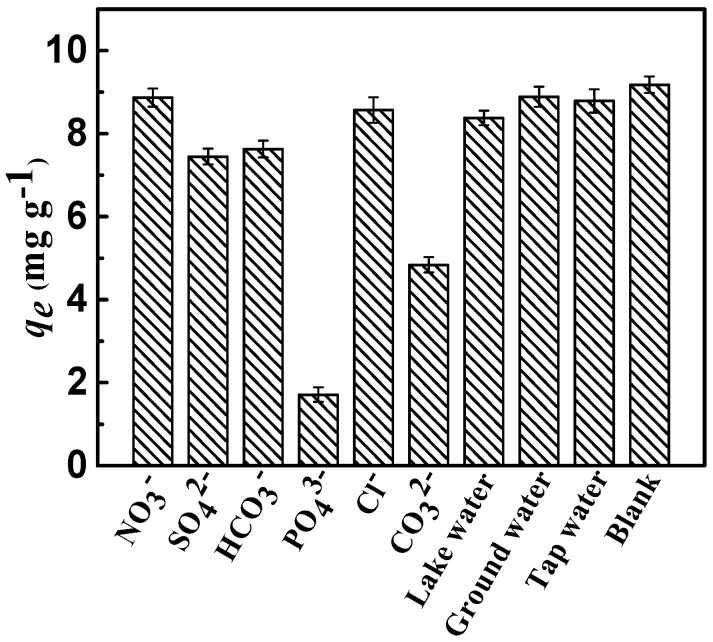
Removal of fluoride as a function of the co-anions (Conditions: initial concentration = 10 mg·L^−1^, dose = 0.75 g·L^−1^, adsorption time = 3 h, adsorption temperature = 298 K, and pH = 7).

The interference resulting from competitive anions on the sorption of fluoride in the actual sewage water is ubiquitous. Therefore, on the premise of equal ionic strengths of the competing anions, the effects of NO_3_^−^, Cl^−^, CO_3_^2−^, SO_4_^2−^, PO_4_^3−^, and HCO_3_^−^ on the sorption of fluoride on the adsorbent were comprehensively investigated. As shown in Figure 8, completely various competing anions patterns of fluoride were exhibited. Evidently, the presence of various competing anions had a different impact on the removal efficiency of fluoride, and the adsorption capacity *q_e_* of fluoride in the presence of anions decreased in the following order:

PO_4_^3−^ > CO_3_^2−^ > SO_4_^2−^ > HCO_3_^−^ > Cl^−^ ≈ NO_3_^−^.

Compared to monovalent anions, divalent and trivalent anions have a greater effect on the removal efficiency of fluoride; the published literature reported similar results [33]. This might be attributed to the high negative charge density of ions, which could create conditions more inviting for fluoride ions by the layered positive charge. The adsorption sites and capacity for fluoride tended to decrease, mostly because competitive anions were introduced on the surface of CSLDO400. Meanwhile, it indicated that monovalent anions had a limited effect on the removal of fluoride. Figure 8 showed that CSLDO400 performed well in fluoride removal in real water samples, indicating that it exhibited considerable potential for the removal of fluoride.

### 2.3. Adsorption Theory Discussion

#### 2.3.1. Adsorption Kinetics

**Figure 9:**
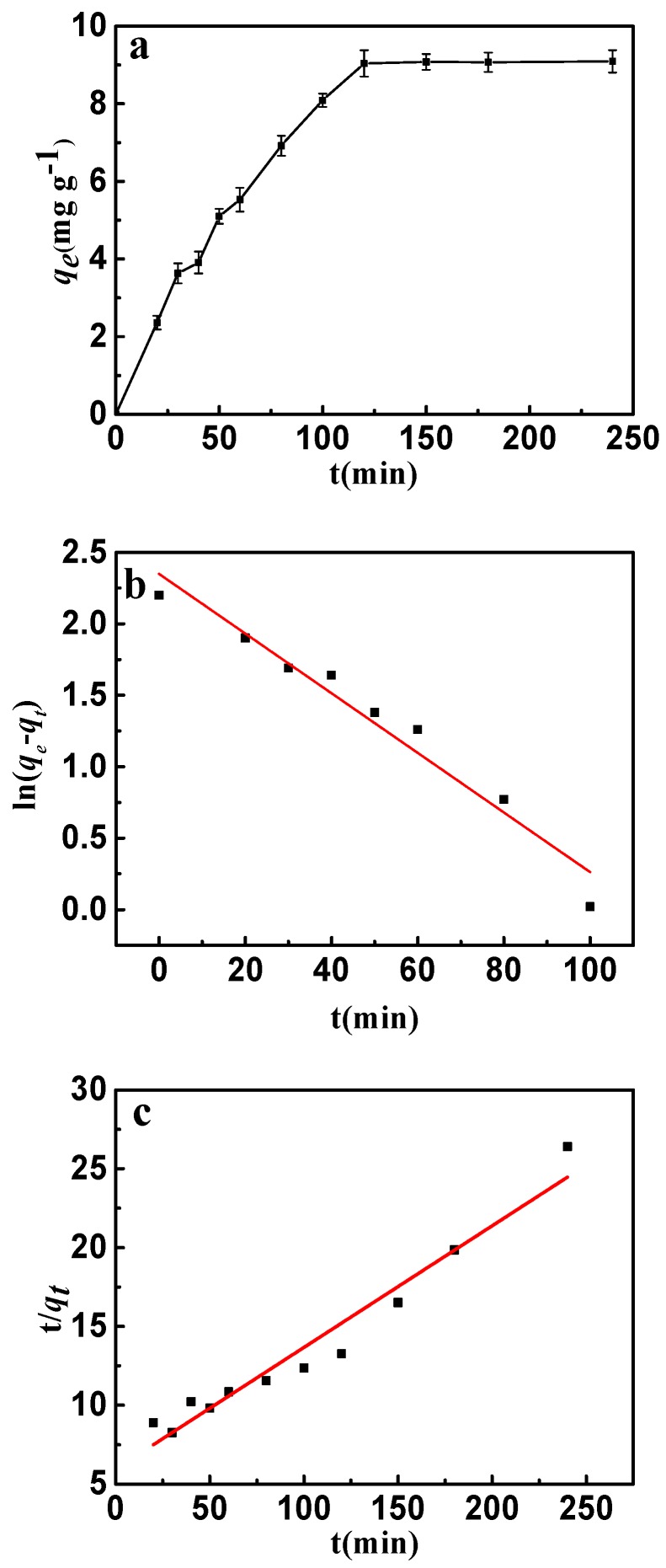
(**a**) Effect of contact time on the adsorption of fluoride on to CSLDO400 (initial concentration, 10 mg·L^−1^; pH = 7; adsorption dose, 0.75 g·L^−1^; temperature, 298 K); (**b**) pseudo-first-order kinetic plots for adsorption of fluoride; (**c**) pseudo-second-order kinetic plots for adsorption of fluoride.

The adsorption kinetics is presented in Figure 9a. It can be seen that the adsorption rate increased rapidly up to 120 min and after that there was no further increase observed. This phenomenon can be attributed to the adsorption and desorption equilibrium. Because of the abundant sorption pores and sites, a fast adsorption rate was discovered in the early stages. Nevertheless, more and more fluorine entering the interlayer of CSLDO400 might result in desorption of fluoride. Desorption rates were almost equivalent to the adsorption rates when the adsorption of fluoride achieved saturation. The adsorption behavior and potential rate-controlling steps were evaluated by pseudo-first-order and pseudo-second-order kinetic models. The kinetic models’ linear forms were given by the following equations [34]:

(4) $$\ln\left(q_{e}-q_{t}\right)=\ln q_{e}-k_{1}t$$

(5) $$\frac{t}{q_{t}}=\frac{t}{q_{e}}+\frac{1}{k_{2}q_{e}{}^{2}},$$

where *k*_1_ and *k*_2_ are the pseudo-first-order and the pseudo-second-order rate constants, *q_t_* (mg·g^−1^) and *q_e_* (mg·g^−1^) are the adsorption capacities at time (*t*) and at equilibrium, respectively. The model fittings are shown in Figure 9b,c, and the kinetic parameters of pseudo-first-order and pseudo-second-order were determined and listed in Table 2. It can be seen from Figure 9b,c that both the pseudo-first-order model and pseudo-second-order model well described the fast adsorption stage. However, as shown in Table 2, the pseudo-first-order model showed a higher correlation coefficient for fluoride compared with the pseudo-second-order model. Evidently, the adsorption of fluoride can be described more appropriately by the pseudo-first-order model.

#### 2.3.2. Adsorption Isotherm

**Figure 10:**
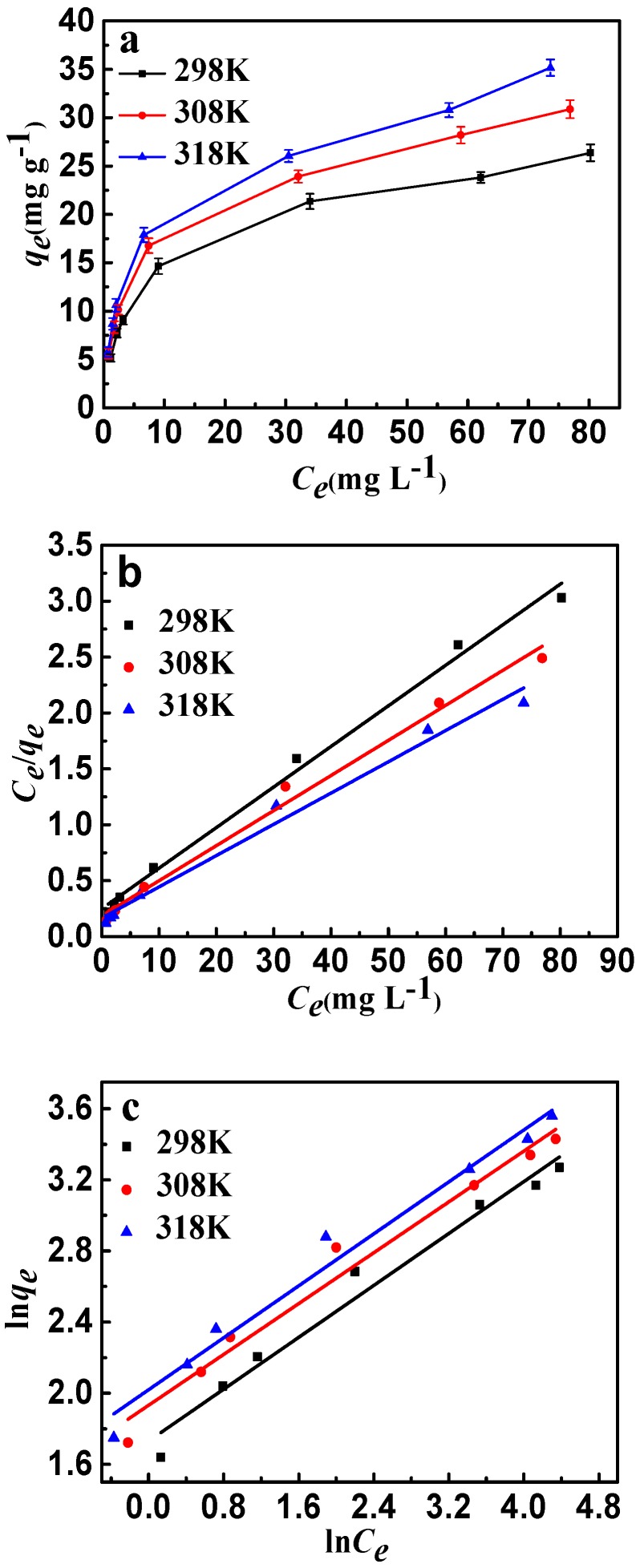
(**a**) Adsorption isotherms of fluoride ion adsorption onto CSLDO400 (Conditions: concentration was 5, 8, 10, 20, 50, 80, 100 mg·L^−1^, pH = 7, dose = 0.75 g·L^−1^, adsorption time = 3 h); (**b**) Langmuir plots of the isotherms for fluoride; (**c**) Freundlich plots of the isotherms for fluoride.

The effects of temperature and initial concentration of fluoride are shown in Figure 10a. For each of the same initial concentrations, the increase of adsorption temperature led to an increase in fluoride adsorption capacity due to the decrease of equilibrium concentration. Langmuir and Freundlich are the most significant isotherm models of adsorption valuation and have been used widely. The Langmuir model is based on the monolayer adsorption occurring on a homogeneous adsorbent surface with identical adsorption sites. The Freundlich model describes the adsorption on an energetically heterogeneous surface. The Langmuir and Freundlich models were represented as follows: [35]

(6) $$\frac{C_{e}}{q_{e}}=\frac{C_{e}}{q_{m}}+\frac{1}{bq_{m}}$$

(7) $$\ln q_{e}=\frac{1}{n}\ln C_{e}+\ln K_{f},$$

where *C_e_* (mg·L^−1^) is the adsorption concentration at equilibrium, *q_m_* (mg·g^−1^) is the maximum adsorption amount, *q_e_* (mg·g^−1^) is the adsorption capacity at equilibrium, *b* is the Langmuir constant, and *K_f_* is the Freundlich constant. The isotherm fittings are shown in Figure 10b,c, and the parameters of the Langmuir and Freundlich models are listed in Table 3. It can be seen that the experimental data of fluoride have a better fit by the Langmuir isotherm model than the Freundlich isotherm model. The results may indicate that the adsorbent type is monolayer and all adsorption sites are energetically equivalent; calcination treatment would play a significant role in the adsorption of fluoride. From the Langmuir isotherm model, the maximum sorption capacity (*q_m_*) of fluoride is 27.56 mg·g^−1^ at room temperature. In addition, the Freundlich constants (*n*) were both greater than 1.0, indicating that CSLDO400 was favorable for the removal of fluoride under the studied conditions.

#### 2.3.3. Adsorption Thermodynamics

In particular, the determination of thermodynamics parameters is widely used to evaluate spontaneity and heat change of the adsorption reactions [36]. The values of entropy change (∆*S*) and enthalpy change (∆*H*) were obtained from the following Van’t Hoff plot:

(8) $$\mathrm{lnb}=\frac{△S}{R}-\frac{△H}{RT}$$

The Gibbs free energy changes (∆*G*) were given by the following equation:

(9) △G=△H−T△S

where *b* is the Langmuir constant. The parameters ∆*S*, ∆*H*, ∆*G* and correlation coefficient (*R*^2^) were described in the Table 4. The enthalpy change of fluoride was 5.706 kJ·mol^−1^, implying that the fluoride adsorption process is endothermic and favored in high-temperature conditions. The positive entropy changes of fluoride (60.52 J·mol^−1^·K^−1^) indicated an increase in the randomness of the irreversible adsorption processes and hence a good affinity of fluoride towards the CSLDO400 particles. The Gibbs free energy changes were also calculated at 298, 308, and 318 to be −12.33, −12.93, and −13.54 kJ·mol^−1^, respectively. The negative values of those ∆*G* indicate the spontaneous nature of the adsorption process.

#### 2.3.4. Adsorption Mechanism

**Figure 11:**
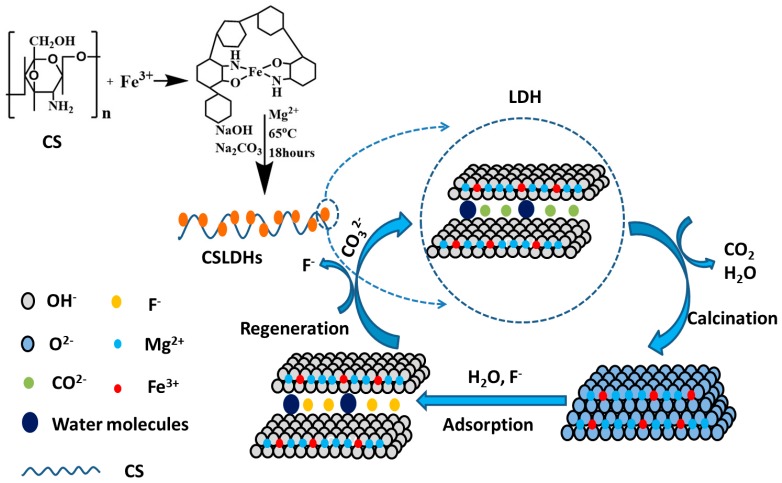
Schematic diagram of the adsorption of fluoride on CSLDO400.

The adsorption process was likely controlled by the “memory effect.” F^−^ could be adsorbed onto CSLDO400 by physical adsorption, chemisorption, and surface coordination. The CSLDHs will lose water molecules and interlayer anions, and generate pores after being calcined at a certain temperature, which contributes to the increase of specific surface area and adsorption sites. CSLDO400 was restored to its original layered structure after entering into a water environment. Mg^2+^ of the structure of CSLDO400 were replaced by Fe^3+^, which can result in a large number of positive charges in the interlayer of CSLDO400. Therefore, the calcined chitosan supported LDHs may be forced to adsorb fluoride ions from the water solution to maintain a balance between charges in the interlayer of CSLDO400. In an aqueous solution, hydroxylate surfaces were created through the coordination reaction between OH^−^ and the uncoordinated metal ions of CSLDO400. After an exchange between F^−^ and OH^−^ of ≡MeOH has taken place, the coordination reaction may ultimately be achieved. The design of the synthetic and adsorption process is shown as Figure 11.

### 2.4. Regeneration and Reuse

It is of great importance to consider the regeneration performance of an adsorbent in practical applications. During the adsorption of fluoride, the layer structure of CSLDO400 was not changed because the F^−^ were just loaded on the interlayer of CSLDO400, and the regeneration of CSLDO400 was achieved easily [37,38]. Considering the co-anions’ effect on the adsorption of fluoride in previous studies, desorption was used in this paper.

**Figure 12:**
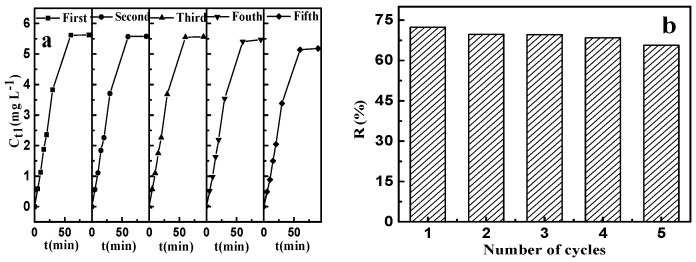
Effect of recycling times on the concentration of fluoride in desorbing solution (**a**); and the removal rate of fluoride (**b**).

The results of adsorption–desorption, which was repeated five times, are shown in Figure 12. It can be seen from Figure 12a that the desorption rate increased rapidly up to 60 min and then no significant change was observed. Comparing to the virgin CSLDO400 with the equilibrium adsorption capacity (*q_e_*) equaling to 9.38 mg·g^−1^ (removal efficiency: 73.91%) for fluoride, the regenerated CSLDO400 showed a slight decrease in removal rate but still exhibited excellent reusability and consistency (as shown in Figure 12b). CSLDO400 have both interconnecting pore channels and a layered structure, which can reduce pore blockage by adsorbate molecules. The affinities of various anions toward interlayers of hydrotalcite, electrostatic interaction between carbonate ions from Na_2_CO_3_ solution, and fluoride ions adsorbed in interlayers of CSLDO400 made the materials achieve good regeneration. The excellent reusability and stability indicate that large-scale and long-term water treatment will be a strong possibility.

### 2.5. Comparison of Fluoride with Other Adsorbents

A comparison has been made between CSLDO400 and other previously reported adsorbents for fluoride removal. Based on the maximum adsorption capacities *q_max_* from the Langmuir isotherm model, CSLDO400 as an adsorbent was calculated to be 27.56 mg·g^−1^ for fluoride at 298 K, and it also showed a wider range of pH and better adsorption for fluoride than many of the adsorbents reported (shown in Table 5). The high adsorption capacity obtained in this work might be attributed to interconnecting pore channels, layered structure, and high affinity between Mg/Fe oxides and fluoride ions. Therefore, it can be concluded that the CSLDO400 adsorbent has considerable potential for the removal of fluoride pollutants from an aqueous solution.

## 3. Experimental

### 3.1. Materials

Mg–Fe LDH was synthesized by the co-precipitation of the nitrate precursors, iron (III) nitrate nonahydrate (Fe(NO_3_)_3_·9H_2_O) and magnesium nitrate hexahydrate (Mg(NO_3_)_2_·6H_2_O) (synthesis grade, purchased all from Sinopharm Chemical Reagent Co., Ltd., Shanghai, China). The chitosan support for the preparation of Mg–Fe LDH (CSLDHs) was prepared from a chitosan solution, and chitosan was obtained from Aladdin. NaF solutions of different concentrations were obtained by dissolving analytical reagent grade sodium fluoride (Shanghai Maikelin Biochemical Co., Ltd., Shanghai, China) in de-ionized water.

### 3.2. Synthesis of Calcined Chitosan Support Layered Double Hydroxides (CSLDO)

The alkali resistance and chelation capacity of transition metal of chitosan are strong, but acid resistance is poor; the chitosan support layered double hydroxide was synthesized by the co-precipitation method. Chitosan solution with a concentration of 4 wt % was prepared by dissolving 3 g chitosan in glacial acetic acid (5 wt %). Solution A was prepared by dissolving Mg(NO_3_)_2_·6H_2_O (0.12 mol) and Fe(NO_3_)_3_·9H_2_O (0.04 mol) in 150 mL deionized water. Solution B was prepared by dissolving NaOH (0.258 mol) and Na_2_CO_3_ (0.08 mol) in 100 mL deionized water. First, solution A was mixed with chitosan solution with stirring for 1 h. Solution B was then slowly added dropwise to the mixture above, stirring violently at 60 °C for 0.5 h, with the solution pH held in the range 11–12. The precipitated mass was aged at 65 °C for 18 h, filtered, washed with distilled water until the filtrate was neutral to litmus, and freeze-dried for 24 h, and chitosan support layered double hydroxides (CSLDHs) were obtained. Three samples, CSLDO300, CSLDO400, and CSLDO500, were obtained after CSLDHs were calcined at 300 °C, 400 °C, and 500 °C under N_2_ protection for 2 h, respectively. Unsupported LDHs were calcined at 400 °C (LDO400) as a comparison.

### 3.3. Sample Characterization

The CSLDHs, unsupported LDO400, CSLDO300, CSLDO400, and CSLDO500, were characterized for their crystallinity, mineralogical phases, morphology, and specific surface area. The morphology of the microspheres was investigated by using a FEI Sirion 200 (FEI Co., Eindhoven, The Netherlands) field emission scanning electron microscope (FEI-SEM) with an accelerating vo ltage of 10 kV. The BET surface area of these samples were determined by N_2_ adsorption–desorption technique on a Micrometrics 2020HD88 (Micrometrics Instrument Co., Norcross, GA, USA) apparatus at 77 K, in which the samples were degassed at 60 °C for 12 h before measurement. The Fourier transform infrared (FT-IR) spectroscopy was measured using Vertex 70 (Bruker, Madison, WI, USA). The mineralogical phases and crystallinity of samples were characterized by X-ray diffraction analyzer (X’Pert PRO MPD) using Cu-Kα radiation.

### 3.4. Adsorption Experiments

A contrasting experiment on the effects of time was performed on the CSLDHs LDO400, CSLDO300, CSLDO400, and CSLDO500. The adsorptions of fluoride were investigated for the effects of adsorbent dosage, solution pH, co-anions, and the adsorption theory (adsorption kinetics, isotherms thermodynamics). Fluoride solutions with different concentrations were obtained by dissolving NaF in deionized water.

In order to decide the optimal adsorbent for the adsorption experiments, CSLDHs LDO400, CSLDO300, CSLDO400, and CSLDO500 with a mass of 25 mg were weighed precisely and added to 40-mL aqueous solutions of fluoride (10 mg·L^−1^) and shaken at 170 rpm at 298 K for 3 h. The fluoride concentration (*C_t_*) in the solution was analyzed using a fluoride ion selective electrode (ISE, Ruosull PF-1Q9, Shanghai Instrument Factory Co., Shanghai, China), every 20 min for 3 h. The adsorption capacity (*q_t_*: mg·g^−1^) of CSLDHs LDO400, CSLDO300, CSLDO400, and CSLDO500 was calculated using Equation (10):

(10) $$q_{t}=\frac{\left(C_{0}-C_{t}\right)\times V}{m}$$

The removal rates (R: %) were calculated using the following equation:

(11) $$R\left(\%\right)=\frac{C_{0}-C_{t}}{C_{0}}\times 100\%$$

where *C*_0_ and *C_t_* (mg·L^−^^1^) are the initial concentrations and concentrations at time *t*, respectively; *V* (L) is the volume of the solution and *m* (g) is the mass of the adsorbent.

In order to optimize CSLDO400 dosages, CSLDO400 with different dosage (0.375, 0.5, 0.625, 0.75, 0.875, and 1.0 g L^−1^) were investigated. The effect of pH was studied in the range of 3.0 to 13.0. To study co-anions, adsorption experiments were carried out in the presence of various competing anions such as NO_3_^−^, Cl^−^, CO_3_^2−^, SO4^2−^, PO_4_^3−^, and HCO_3_^−^ by taking equal ionic strengths to that of the fluoride solution (0.526 mmol·L^−1^). Real fluoride-contaminated water samples were obtained by dissolving a certain amount of NaF in river water (Beijing, China), groundwater (Beijing, China), and surface drinking water (from the third drinking water treatment plant in Beijing, China), respectively. The concentrations of fluoride in all the real water samples were obtained with 10 mg·L^−1^. Major anion contents in real water are listed in Table 6. To study the kinetics, CSLDO400 (0.75 g·L^−1^) was mixed with a fluoride solution (10 mg·L^−1^) and tested over a certain time interval. The adsorption isotherms were finished with different initial concentrations (5, 8, 10, 20, 50, 80, and 100 mg·L^−1^). The thermodynamic features were studied by using isothermal adsorption experiments that were repeated at 298 K, 308 K, and 318 K. The adsorbed CSLDO400 was regenerated: the fluoride-adsorbed CSLDO400 was eluted by sodium carbonate solution (0.5 mol·L^−1^), and then filtered, washed with distilled water until the filtrate was neutral to litmus, and calcined at 400 °C for 2 h.

## 4. Conclusions

In this work, calcined chitosan-supported layered double hydroxides were successfully synthesized by the co-precipitation method. The adsorption tests suggested that the optimal calcination temperature was 400 °C. The as-prepared CSLDO400 exhibited a porous and layered structure and the largest surface area, resulting in excellent adsorption performance towards fluoride (the maximum adsorption capacity and the adsorption equilibrium times at 298 K are about 27.56 mg·g^−1^ and 120 min). The fluoride removal by CSLDO400 followed the pseudo-first-order model and Freundlich isotherm; the adsorptions of fluoride ions were spontaneous and endothermic. In addition, excellent regeneration performance was obtained in reuse experiments. Moreover, the fluoride removal rate of the low concentration in fluoride solution (5 mg·L^−1^) reached 77%, and the equilibrium concentration was 1.15 mg·L^−1^, which is below the WHO guidelines.

## Figures and Tables

**Table 1:** Specific surface area and pore volume parameters of CSLDHs, LDO400, CSLDO300, CSLDO400, and CSLDO500.

Materials	S_BET_^a^/m^2^·g^−1^	S_mic_^b^/m^2^·g^−1^	V_mic_^c^/cm^3^·g^−1^	V_meso_^d^/cm^3^·g^−1^	V_t_^e^/cm^3^·g^−1^	D_p_^f^/nm
CSLDHs	16.38	2.03	0.0003	0.0557	0.056	13.28
LDO400	80.73	7.59	0.0056	0.3174	0.323	15.83
CSLDO300	47.55	4.05	0.0012	0.1538	0.155	12.01
CSLDO400	116.98	4.37	0.0014	0.4106	0.412	8.84
CSLDO500	94.35	2.21	0.0005	0.3175	0.318	10.99

^a^ Determined by N_2_ adsorption using the Brunauer–Emmett–Teller (BET) method; ^b^ Micropore area, determined by DFT; ^c^ Micropore volume, calculated using the Dubinin–Astakhov method; ^d^ Mesopore volume, calculated by V_t_ − V_mic_; ^e^ Total pore volume, determined at P/P_0_ = 0.9923; ^f^ Adsorption average pore width (4 V/A by BET).

**Table 2:** Parameters for fluoride adsorption by CSLDO400 according to different kinetic models.

*q_e_*_(exp)_ (mg·g^−1^)	Pseudo-First-Order Kinetic Model			Pseudo-Second-Order Kinetic Model		
	*k*_1_ (min^−1^)	*q_e_*_1(cal)_ (mg·g^−1^)	*R*^2^	*k*_2_ (×10^−4^) (g·mg^−1^·min^−1^)	*q_e_*_2(cal)_ (mg·g^−1^)	*R*^2^
9.58	0.02	10.47	0.9514	9.99	12.97	0.9483

**Table 3:** Isotherm model constants and correlation coefficients for adsorption of fluoride onto CSLDO400 at different temperature.

*T* (K)	Langmuir Model			Freundlich Model		
	*b* (L·mg^−1^)	*q_max_* (mg·g^−1^)	*R*^2^	*n*	*K_f_*	*R*^2^
298	0.1457	27.56	0.9930	2.740	5.6423	0.9724
308	0.1689	31.88	0.9909	2.797	6.9023	0.9735
318	0.1695	35.77	0.9842	2.7372	7.5334	0.9756

**Table 4:** Thermodynamic parameters for the adsorption of fluoride.

*T* (K)	∆*S* (J·mol^−1^·K^−1^)	∆*H* (kJ·mol^−1^)	∆*G* (kJ·mol^−1^)	*R*^2^
298	60.52	5.706	−12.33	0.9576
308	-	-	−12.93	-
318	-	-	−13.54	-

**Table 5:** Comparison of adsorption capacity of CSLDO400 with different adsorbents.

Adsorbents	*q_max_* (mg·g^−1^)	pH	References
CSLDO400	27.56	5~9	Present study
Iron–aluminum mixed oxide	17.73	5.5~5.7	[39]
Quick lime	16.67	-	[40]
CSLDH-75	13.8	-	[13]
Granular ceramic	12.12	5~8	[41]
Ceramic adsorbent	2.16	5.8 ± 0.2	[42]

**Table 6:** Major anion contents in real water samples.

Samples	NO_3_^−^ (mg·L^−1^)	SO_4_^2−^ (mg·L^−1^)	HCO_3_^−^ (mg·L^−1^)	PO_4_^3−^ (mg·L^−1^)	Cl^−^ (mg·L^−1^)	CO_3_^2−^ (mg·L^−1^)
Lake water	110.97	62.65	312.78	detection limit	84.41	35.62
Tap water	5.87	53.71	225.37	detection limit	68.16	10.23
Groundwater	15.49	38.59	301.74	detection limit	71.36	27.38

Note: detection limit means that PO_4_^3−^ concentration was not detected because they were below the detection limit of this detection method.
